# Utility of Next-Generation Sequencing-Based Chimerism Analysis for Early Relapse Prediction following Allogenic Hematopoietic Cell Transplantation

**DOI:** 10.3390/ijms25052811

**Published:** 2024-02-28

**Authors:** Heerah Lee, Seung-Won Chae, Sung Im Cho, Jee-Soo Lee, Man Jin Kim, Moon-Woo Seong

**Affiliations:** 1 Department of Laboratory Medicine, Chosun University Hospital, Gwangju 61453, Republic of Korea; heerah@hotmail.com; 2 Cancer Research Institute, Seoul National University College of Medicine, Seoul 03080, Republic of Korea; 3 Department of Laboratory Medicine, Seoul National University Hospital, Seoul National University College of Medicine, Seoul 03080, Republic of Korea; 20573@snuh.org (S.I.C.);; 4 Department of Genomic Medicine, Seoul National University Hospital, Seoul 03080, Republic of Korea; sesrajarus@hanmail.net

**Keywords:** hematopoietic cell transplantation, chimerism, next-generation sequencing, short tandem repeat, relapse, monitoring

## Abstract

Chimerism monitoring following allogeneic hematopoietic cell transplantation (HCT) plays a pivotal role in evaluating engraftment status and identifying early indicators of relapse. Recent advancements in next-generation sequencing (NGS) technology have introduced AlloSeq HCT as a more sensitive alternative to short tandem repeat (STR) analysis. This study aimed to compare AlloSeq HCT with STR, focusing on the prediction of early relapse post-allogeneic HCT. Chimerism levels in 29 HCT recipients were assessed using both STR and NGS, employing a total of 125 whole blood or bone marrow aspirate samples (68 post-HCT and 57 pre-HCT samples from recipients or donors). AlloSeq HCT exhibited high concordance with STR and demonstrated the potential for early detection of chimeric changes, particularly at extremely low levels. The combined advantages of high sensitivity and automated data analysis offered by AlloSeq HCT substantiate its clinical adoption for effective chimerism monitoring.

## 1. Introduction

Allogeneic hematopoietic cell transplantation (HCT) stands as a curative therapeutic modality for both malignant and non-malignant disorders [1]. The increasing number of HCT procedures and enhanced survival rates have brought about several ongoing challenges, including engraftment failure, graft-versus-host disease (GVHD), and disease relapse [1]. Minimal residual disease (MRD) analysis of gene fusions or pathogenic variants offers substantial prognostic insights post-HCT. Simultaneously, the assessment of the patient-to-donor cell ratio, known as chimerism monitoring, serves to confirm donor cell engraftment. Moreover, chimerism monitoring is suggested for detecting potential relapses, especially in cases lacking suitable MRD markers [2,3]. Amanda et al. [4] reported that 69% of the members of the Center for International Blood and Marrow Transplant Research use chimerism testing for detecting impending relapse, with most of them emphasizing engraftment monitoring as the primary indication. Chimerism analysis could potentially impact treatment decisions, thereby influencing the choices related to donor lymphocyte infusion, immunosuppression, chemotherapy, and second HCT [4,5]. However, there are no established guidelines for chimerism analysis concerning clinical indications, testing time points, duration of follow-up, and specimen sources. Additionally, consensus is lacking on the clinically significant threshold of chimerism level or changes over time [4,6].

Various methods can be employed for chimerism analysis utilizing polymorphic markers to differentiate donor cells from recipient cells. These methods encompass chromosome analysis, fluorescence in situ hybridization (FISH), short tandem repeat (STR) PCR, quantitative PCR (qPCR), digital PCR (dPCR), and next-generation sequencing (NGS) [2,7,8]. Each method has its strengths and weaknesses in terms of sensitivity, precision, applicability, and cost-effectiveness. STR-PCR, regarded as the gold standard method, has widespread application due to the availability of commercial kits and extensive experience. PCR followed by fragment analysis of a limited number of multiallelic markers provides advantages such as a short turnaround time, high informativeness, and sufficient sensitivity, with a limit of detection (LOD) of >1% [9]. Nevertheless, STR-PCR encounters technical limitations, including stutter peaks and amplification bias, necessitating manual interpretation of results and analyst expertise [10]. An LOD of 1% is often insufficient to detect a relapse before clinical manifestation [11]. Novel and more sensitive methods (featuring LODs of 0.01 to 1%), such as dPCR, qPCR, and NGS, have the potential to facilitate early relapse detection, allowing for timely intervention [5]. NGS, characterized by high precision and sensitivity, surpasses qPCR in precision and STR in sensitivity.

Persistent donor chimerism does not necessarily indicate an incipient relapse or engraftment failure [5]. Chimerism undergoes physiological fluctuations and is influenced by GVHD, immunosuppressive therapy, viral infections, and various other factors [11]. A progressive increase over time provides more informative insights [4,12]. Therefore, the timely detection of low-level chimerism is crucial at a single time point, requiring high sensitivity and precision to reliably detect minute changes over time.

In the present study, we assessed the AlloSeq HCT kit (CareDx, Brisbane, CA, USA) for allogeneic HCT chimerism monitoring and the detection of impending relapse by comparing its results with those obtained using STR-PCR.

## 2. Results

### 2.1. Patients and Samples

The indications for HCT in the study included hematologic malignancies, including twelve acute lymphocytic leukemia (ALL; 12/29, 41%), eight acute myeloid leukemia (AML; 8/29, 28%), and three non-hematologic malignancies. Various types of HCT were included (59% related and 41% unrelated). The level of HLA matching varied from haploidentical to partially and fully matched. The interval between consecutive tests was 28 days (median, range 3–48) and 118 days (median, 15 days–56 months) after HCT. Among the post-HCT samples, 60% (41/68) exhibited complete chimerism (CC) with <0.8% STR results. Overall, 12 relapses were observed, with a relapse defined as ≥5% blasts in bone marrow by morphology or flow cytometry (Table 1 and Table 2).

The median of initial DNA concentration was 8.16 ng/µL (range 0.90–50) by Qubit 3.0 fluorometer (Life Technologies, Carlsbad, CA, USA). The final library concentration was 0.26~0.265 ng/µL by Qubit and within the 250–300 base pair range by 4200 TapeStation system (Agilent, Santa Clara, CA, USA). The median of total read depths was 856,389 (range 198,812–2,416,888), and the average number of reads covering each marker was 1871.43 (range 383.6–5558.1).

### 2.2. Utilization of Donor–Recipient Genotype Information Regarding AlloSeq HCT

The post-HCT sample could be analyzed in two modes—blind and targeted. The latter utilizes the genotype of contributors in various combinations. Therefore, blind, recipient-targeted, donor-targeted, and double-targeted analyses could be performed utilizing no, recipients’, donors’, and both pre-HCT genotypes, respectively. The results from the three types of targeted genotypes revealed a high correlation with a small difference (pooled standard deviation (SD): 1.54). The results also exhibited a significant correlation with the STR results, even in cases in which the quality control (QC) yielded a failure or warning.

In contrast, in cases of more than one donor, genetic contributors for targeted post-HCT sample analysis should be carefully selected. Table 3 summarizes the chimerism levels depending on the genotype utilized in post-HCT analysis of patient 20. He received HCT from two different HLA-matched sisters. At post-1 HCT, STR showed the CC of donor 2. When donor 1 genotype was selected rather than that of donor 2, the chimerism value was calculated to be absurdly high.

### 2.3. Informativeness of AlloSeq HCT

Twenty-four percent (7/29) of patients had a complex karyotype (≥3 abnormalities), which complicated STR-PCR analysis and reduced the number of informative markers. The minimum of three markers required for accurate analysis were not secured in 10% (7/68) of post-HCT STR monitoring cases.

AlloSeq HCT disregards the potential presence of chromosomal abnormalities in post- and pre-HCT samples, thereby exploiting 202 single nucleotide polymorphisms (SNPs) and the multiplexing nature of NGS. In total, 102 markers (median, range 63–136) were considered informative, securing a higher number of markers than those achieved with STR. For unrelated recipient/donor pairs, 123 (111–136) markers were informative, whereas 83 (63–113) were informative for related pairs. Regarding the chimerism level, for those samples above 0.8%, 109 (63–136) markers were informative, and for those below 0.8%, 100 (63–136) were informative. Patients with complex karyotypes had 102 (80–136) informative markers, while patients without complex karyotypes had 101 (63–130).

### 2.4. Correlation between the Results of AlloSeq HCT and STR in Determining Chimerism Level

**Figure 1:**
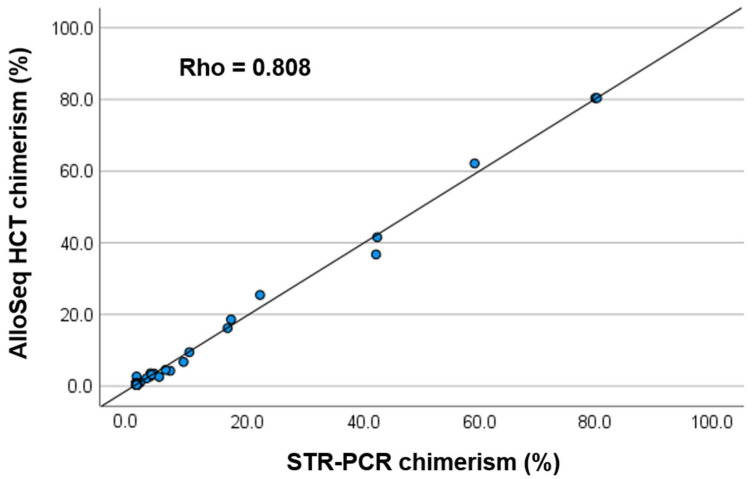
Correlation between % chimerism after allogeneic hematopoietic cell transplantation, assessed using short tandem repeat (STR)-PCR, and AlloSeq HCT in 68 post-transplant samples.

**Figure 2:**
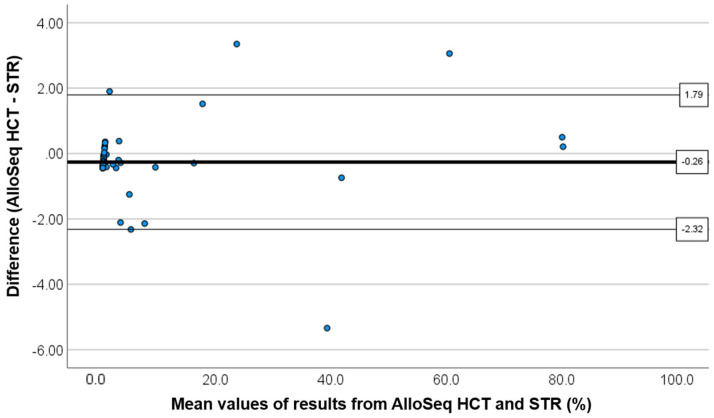
Bland–Altman analysis comparing % chimerism after allogeneic hematopoietic cell transplantation, assessed using short tandem repeat (STR)-PCR and AlloSeq HCT. The plot includes the mean values (thick solid line) and the ±2 standard deviation values (thin solid lines), with absolute values depicted in boxes.

The AlloSeq HCT was performed for 68 post-HCT samples. Of 48 samples that tested positive for CC with the STR assay, 26 exhibited detectable mixed chimerism (MC) (>0.36%) according to the AlloSeq HCT results; their level was 0.41–2.7 (0.77 ± 0.90; mean ± 2SD). A correlation study of all 68 samples showed a high correlation between the results obtained using STR and AlloSeq HCT (rho = 0.808, Spearman’s rank test) (Figure 1). The Bland–Altman plot did not reveal any constant or proportional errors (Figure 2). Twenty MC samples confirmed via STR (>0.8%) showed a higher correlation between the results obtained using STR and AlloSeq HCT (rho = 0.977).

### 2.5. Value of Kinetic Chimerism Approach for Predicting Relapse

**Figure 3:**
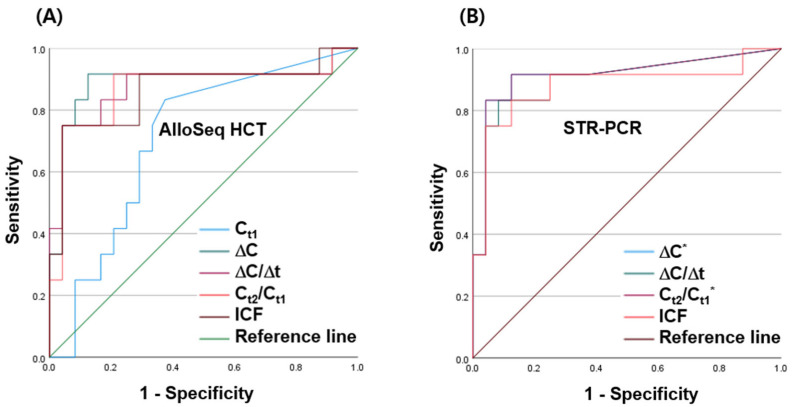
Receiver operating characteristic (ROC) curves depicting % chimerism for the prediction of relapse using (**A**) AlloSeq HCT and (**B**) short tandem repeat (STR)-PCR. * The blue line is fully coincident with the purple line.

We assessed the diagnostic value of the highly sensitive AlloSeq HCT in predicting relapse or adverse clinical outcomes. Receiver operating characteristic (ROC) analysis was employed to evaluate the performance of AlloSeq HCT in predicting impending relapse by measuring parameters such as Ct1, ΔC, ΔC/Δt, Ct2/Ct1, and ICF. The area under the curve (AUC) values and 95% confidence intervals for these parameters were as follows: 0.707 (0.530–0.883), 0.889 (0.740–1.000), 0.875 (0.725–1.000), 0.868 (0.715–1.000), and 0.861 (0.711–1.000), respectively. Among these, ΔC and Ct2/Ct1 of STR demonstrated the largest AUC, at 0.911 (0.791–1.000) (Figure 3). Ct1 of AlloSeq HCT showed the lowest AUC. Based on the ROC curves, ΔC > 2.36 was the mutually optimal cut-off for AlloSeq HCT and STR when sensitivity and specificity were optimized. The sensitivity, specificity, positive predictive value, and negative predictive value for relapse prediction were calculated (Table 4). Despite STR showing higher AUC for most predictors (except Ct1 due to the study design), AlloSeq HCT demonstrated comparable capability for relapse detection.

## 3. Discussion

**Figure 4:**
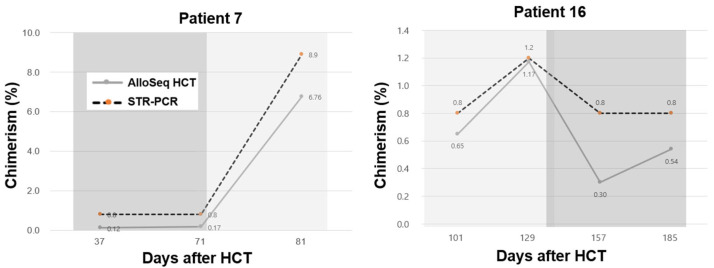
Chimerism trajectories of Patients 7 and 16. Changes in chimerism (light gray) and no changes in chimerism (dark gray) detected using short tandem repeat (STR)-PCR.

AlloSeq HCT demonstrated predictive capabilities comparable with those of STR-PCR for relapse detection. The seemingly paradoxical result of AlloSeq HCT, wherein its superior sensitivity does not translate into greater predictive power for relapse, could be explained through a detailed examination of specific patient cases. For instance, Patient 7 exhibited the potential for early intervention; in this patient, an increase in chimerism from 0.12% to 0.17% was detected using the sensitive AlloSeq HCT (Figure 4). Similarly, Patient 17 showed an escalation in chimerism levels from 0.41% and 0.67% to 80.41%, which was not detected using STR (0.8%, 0.8%, and 80.2%) (Table 2). These cases highlight the transition detection capability of NGS, especially at extremely low chimerism levels. Further investigation is needed to determine whether a ΔC of 0.05% represents a significant change. In contrast, the case of Patient 16 supports the notion of overly sensitive methods detecting physiological fluctuations, potentially raising false alarms of relapse or engraftment failure (Figure 4). Meanwhile, Patient 27 experienced relapse despite being categorized in the non-relapse (NC) group, illustrating a negative ΔC, as assessed using AlloSeq HCT (–0.12% = 0.95–0.83%). Notably, 35% (7/20) of patients in the CC group did not experience relapse, which underscores the complex relationship between chimerism and disease relapse, necessitating a critical reevaluation of the utility of chimerism monitoring as a reliable predictor of relapse, irrespective of the detection method. Determining predictors of the changes in chimerism and their significance over time warrants further exploration.

The ideal method for chimerism monitoring should be sensitive, precise, cost-effective, rapid, easy to perform/interpret, and universally applicable [6]. AlloSeq HCT demonstrated higher sensitivity than STR-PCR, enabling the quantification and detection of small changes at low chimerism levels (<0.8%). The sensitivity of NGS could be improved further to 0.01% by means of increasing the DNA input or lineage specific analysis. The need for increased sensitivity in chimerism detection is a topic of debate, primarily due to concerns about potential false-positive results [6]. Moreover, increased sensitivity amplifies the visibility of chimerism fluctuations [11], which could arise from contamination by the recipient’s skin, epithelial cells, or stromal cells during sample collection [4]. This variation may also be influenced by the different types of post-HCT samples, ranging from the bone marrow aspirate (BMA) to whole blood (WB). Nevertheless, there is a general consensus that an increase in chimerism levels and timely detection of changes expand the window for therapeutic intervention [4]. NGS is costly in terms of both initial equipment setup and consumables. The high expense impedes frequent testing, thus hampering the realization of its superior sensitivity and early relapse detection capability.

AlloSeq HCT holds an advantage not only in sensitivity (with its lower LOD and limit of quantitation (LOQ) than STR) but also in precision. The order for increasing levels of imprecision is as follows: NGS, STR (coefficient of variation (CV): 1–20%), and qPCR (CV: 30–50%) [1,4,12]. AlloSeq HCT requires less genetic material for testing and utilizes a greater number of markers without the need for the genotypes of all contributors. Several researchers have prepared artificial samples with different chimerism values by mixing two samples in different proportions to verify the performance of AlloSeq HCT. It is essential to note that artificial samples cannot fully substitute for the clinical post-HCT samples from patients. Liacini et al. [2] calculated the overall CV as 5.08% (ranging from 0% to 11.21% at the expected chimerism value of 0.1–10%), and Janakiraman et al. [13] demonstrated within- and across-run variability of 4.7% and 4.1%, respectively. Additionally, Picard et al. documented a lower LOQ (0.22%) than that reported by the manufacturer (Table 5) [8].

Additional benefits of AlloSeq HCT include its automated and user-friendly chimerism calculations [2]. The presented results encompass the percentage of DNA from each genetic contributor, QC status with reasons, the number of informative markers, and average marker coverage. We strongly recommend conducting analyses with different combinations of pre-HCT genotypes and thoroughly reviewing all QC statuses, especially when inadequate data have been generated during the preparation and sequencing of any sample included in the analysis. In situations in which the pre-HCT genotype sample experiences QC failure or warning, the relevant genotype could be omitted from the post-HCT analysis without impairing the final result. The detection and quantification of low levels of chimerism (<10%) are particularly influenced by the sequencing depth, sample quality, and quantity.

Our study has some limitations. One limitation is the retrospective design, which features inherent biases. Another limitation is the small sample size. In the present study, all samples were tested once, and the values were compared with the chimerism values obtained using the less sensitive STR method, thereby limiting a statistical comparison. Future studies should conduct lineage-specific and cell subset analyses, which require less genomic DNA (gDNA) input and offer higher sensitivity [4]. Calculation methods other than delta or ICF can be developed for the kinetic approach. These limitations provide avenues for future research and development in the field of post-HCT chimerism analysis.

## 4. Materials and Methods

### 4.1. Patients

STR results obtained during January 2014 to May 2023 were reviewed, and all patients with STR follow-up intervals within 50 days were enrolled in the study. Twenty-nine recipient/donor pairs and 125 (68 post-HCT and 57 pre-HCT samples from the recipient or donor) WB or BMA samples were selected. Among all consecutive result pairs, those that changed from negative to positive (recipient chimerism > 0.8%) results were selected as the change in the chimerism (CH) group, while those with two negative consecutive result pairs were selected as the no change in chimerism (NC) group. Pairs of results indicating decreasing chimerism were excluded from the analysis. Finally, 20 CH and 16 NC pairs were selected. We investigated the indication, type, and other characteristics of HCT, as well as the presence of clinical events, such as relapse, in each patient. This study was approved by the Institutional Review Board of Seoul National University Hospital, Korea (approval number: 2212-113-1389). Informed consent for genetic testing was obtained from the patients or their legal guardians.

### 4.2. DNA Isolation

The gDNA was extracted from either BMA or WB and collected in ethylenediaminetetraacetic acid tubes following the manufacturer’s protocol (QIAsymphony SP, Qiagen, Venlo, Netherlands). The residual samples after clinical STR analysis were archived at −40 °C until tested using AlloSeq HCT. The stored samples were thawed and quantified using a NanoDrop 2000/2000C spectrophotometer (Thermo Fisher Scientific, Waltham, MA, USA). The purity of the extracted DNA was assessed and considered good if the absorbance ratio at 260/280 was within 1.8 to 2.1 and the absorbance ratio at 260/230 was within 1.9 to 2.1.

### 4.3. STR Analysis

STR-PCR was performed using the AmpFLSTR Identifier PCR Amplification Kit (Applied Biosystems, Foster City, CA, USA) according to the manufacturer’s instructions. Fragment analysis was conducted using an ABI 3730xl capillary electrophoresis instrument (Applied Biosystems). The percentage of recipient DNA was quantified, using GeneMapper Software 6 (Applied Biosystems), from the individual proportions of recipient peak areas relative to the sum of all signals from each pair of informative STR markers. A chimerism level of <0.8% was set as a laboratory-verified LOD.

### 4.4. AlloSeq HCT Analysis

AlloSeq HCT is a chimerism assay based on targeted NGS and utilizes 202 SNP loci across 22 autosomal chromosomes. The library was prepared according to the manufacturer’s instructions, starting with 10 ng gDNA as the input. Sequencing was performed using MiSeqDx (Illumina, San Diego, CA, USA) and the MiSeq Reagent Kit v3 with 150 cycles. AlloSeq HCT software version 2 was used to quantify chimerism values from the FASTQ files. The AlloSeq HCT software can simultaneously utilize up to three genetic contributors (recipient and two donors) present in the post-HCT samples. The percentage of DNA was determined based on the fraction of different nucleotides sequenced at each informative SNP locus. An informative locus was defined as one of the following: heterozygous in one genotype and identically homozygous in all other genotypes; and homozygous in one genotype and oppositely homozygous in all other genotypes.

### 4.5. Kinetic Analysis of Chimerism Level

Recipient chimerism was analyzed in terms of absolute values and relative changes over time. The relative changes were described using four predictors—ΔC, ΔC/Δt, Ct2/Ct1, and Increment Factor (ICF) (modified from [11]). Ct1 and Ct2 represent the absolute values of chimerism at two consecutive time points, t1 and t2, where t2 immediately follows t1. The time (t) was measured in days after HCT when STR-PCR was performed. The predictors were calculated using the following equations, with Ct1 and Ct2 measured using STR and AlloSeq HCT:

ΔC = Ct2 − Ct1

Δt = t2 − t1

ICF = Δt√(Ct2/Ct1)

A chimerism value of < 0.36% was set as CC according to the manufacturer’s claim of the LOQ for analysis.

### 4.6. Statistical Analysis

Statistical analysis was performed using IBM SPSS Version 25 (SPSS Inc., Chicago, IL, USA) or Excel 2016 (Microsoft Corporation, Redmond, WA, USA). Statistical significance was set at *p* < 0.05.

## 5. Conclusions

This study evaluated the clinical utility of AlloSeq HCT in monitoring chimerism after allogeneic HCT. The efficacy of AlloSeq HCT was comparable with that of STR-PCR in predicting relapse. The higher sensitivity of AlloSeq HCT coupled with automated data analysis capabilities suggests its potential to replace other chimerism monitoring methods. Although our study had certain limitations, our results underscore the potential utility of NGS-based chimerism monitoring in post-HCT settings. In summary, AlloSeq HCT holds great promise as a sensitive and automated tool for chimerism monitoring, with the potential to enhance patient care and treatment decision-making.

## Figures and Tables

**Table 1:** Clinical data of HCT recipients.

ID	Gender	Age (Years) *	Indication of HCT	Type of HCT	Donor	Chimerism ^#^	Outcome ^†^	Means of MRD Monitoring	Chromosomal Abnormality
#1	M	3	Severe aplastic anemia	HLA haploidentical related	M, father	CC	-	None to follow	
#2	M	64	MDS-EB-1	uPBSCT	M, unrelated	MC	Transformation to pure erythroid leukemia	Chromosome analysis	Complex karyotype
#3	M	54	MDS-MLD	HLA identical related	M, brother	CC	-	Chromosome analysis and FISH	del(20q)
#4	M	6	AML evolved from JMML	HLA haploidentical related	F, mother	MC	Expired	Chromosome analysis, FISH, and flow cytometry	
#5	M	15	Relapsed precursor B-ALL	HLA haploidentical related	F, mother	MC	Persistence	Chromosome analysis, FISH, flow cytometry, and quantitative PCR	Complex karyotype
#6	M	15	B-ALL with *BCR*-*ABL1*	HLA haploidentical related	M, father	CC	CNS involvement	Quantitative PCR	
#7	M	7	Relapsed precursor B-ALL with *RUNX1*	HLA haploidentical related	F, sister	MC	Expired	Chromosome analysis, FISH, and flow cytometry	
#8	M	11	Relapsed precursor B-ALL	HLA haploidentical related	M, father	CC	-	FISH, flow cytometry, and quantitative PCR	Complex karyotype
#9	M	17	T-MN (osteosarcoma)	HLA haploidentical related	M, father	MC	Expired	Chromosome analysis and FISH	
#10	M	9	Refractory precursor B-ALL	HLA haploidentical related	F, sister	MC	Expired	Chromosome analysis, FISH, and flow cytometry	Complex karyotype
#11	M	13	Relapsed precursor B-ALL	HLA haploidentical related	M, brother	CC	Relapsed, 2nd different donor uPBSCT	Chromosome analysis and flow cytometry	der(12)t(3;12)
#12	F	27	MPAL, T/Myeloid	HLA mismatched unrelated	M, unrelated	MC	-	Chromosome analysis, FISH, and flow cytometry	Complex karyotype
#13	F	9	MPAL, B/myeloid	uPBSCT	F, unrelated	CC	-		
#14	M	23	Chronic granulomatous disease	uPBSCT	M, unrelated	CC	-	None to follow	
#15	F	13	Relapsed AML s/p uPBSCT	uPBSCT	M, unrelated	MC	Expired	Chromosome analysis, FISH, and flow cytometry	Complex karyotype
#16	F	15	B-ALL with *ETV6*-*RUNX1*	uPBSCT	F, unrelated	CC	-		
#17	M	5	B-ALL with *ETV6*-*RUNX1*	HLA haploidentical related	M, brother	CC	2nd different donor uPBSCT	FISH and flow cytometry	
#18	F	58	AML	HLA matched unrelated	M, unrelated	MC	Expired		
#19	M	12	Relapsed B-ALL	HLA haploidentical related	M, father	MC	Expired	Chromosome analysis, flow cytometry, and quantitative PCR	Complex karyotype
#20	M	41	Relapsed acute monocytic leukemia s/p rHCT	1st: HLA identical related2nd: HLA identical related	1st: F, sister2nd: F, sister	CC	-		
#21	M	45	Peripheral T-cell lymphoma	HLA haploidentical related	F, sister	CC	Expired		
#22	M	16	Relapsed B-ALL with *BCR*-*ABL1*	HLA haploidentical related	M, brother	CC	Expired	Quantitative PCR	
#23	F	16	T-MN (Ewing Sarcoma)	HLA haploidentical related	F, mother	CC	-		
#24	M	40	Acute myelomonocytic leukemia	HLA matched unrelated	F, unrelated	CC	Expired		
#25	M	6	B-ALL	uPBSCT	F, unrelated	CC	-		
#26	F	5	Fanconi anemia	uPBSCT	M, unrelated	CC	-		
#27	F	42	Relapsed B-ALL with *TCF3*-*PBX1*	uPBSCT	M, unrelated	MC	-	FISH	
#28	M	55	MDS-EB-2	HLA haploidentical related	F, sister	CC	Expired		
#29	M	68	AML with MRC	uPBSCT	F, unrelated	CC	-		

Abbreviations: ID, patient identification number; STR, short tandem repeat; CC, complete chimerism; uPBSCT, unrelated peripheral blood stem cell transplantation; MC, mixed chimerism; HCT, hematopoietic cell transplantation; MRD, minimal residual disease; FISH, fluorescence in situ hybridization; M, male; F, female; CNS, central nervous system. MDS, myelodysplastic syndrome; EB, excess blasts; MLD, multilineage dysplasia; AML, acute myeloid leukemia; JMML, juvenile myelomonocytic leukemia; ALL, acute lymphoblastic leukemia; T-MN, therapy related myeloid neoplasms; MPAL, mixed-phenotype acute leukemia; MRC, myelodysplasia-related changes. * Age at last STR follow-up. ^#^ Complete or mixed chimerism at last STR follow-up. ^†^ Overall outcome or clinical condition at last follow-up.

**Table 2:** Post-HCT sample pairs used for predicting early relapse.

Patient ID and Sample Pairs	STR Chimerism (%)	AlloSeq Chimerism (%)	Interval between HCT and Ct1 (Days)	Interval between Ct1 and Ct2 (Days)	Relapsed
#1 post1–post2	0.8; 79.9	0.48; 80.40	23	11	Yes
#2 post1–post2	0.8; 22.1	0.73; 25.45	28	3	Yes ^#^
#3 post1–post2	0.8; 3.2	0.36; 3.58	77	28	No
#3 post3–post4	0.8; 0.8	0.6; 0.36	143	33	No
#4 post1–post2	0.8; 0.8	1.16; 0.55	124	28	No
#4 post2–post3	0.8; 3.8	0.55; 3.52	142	28	Yes
#5 post1–post2	0.8; 0.8	0.74; 0.72	989	36	No
#5 post2–post3	0.8; 42.1	0.72; 36.76	1025	26	No
#6 post1–post2	0.8; 3.1	0.36; 2.66	453	10	No
#7 post1–post2	0.8; 0.8	0.36; 0.36	37	34	No
#7 post2–post3	0.8; 8.9	0.36; 6.76	71	10	Yes
#8 post1–post2	0.8; 6.6	0.62; 4.28	574	28	Yes ^##^
#9 post1–post2	0.8; 5.8	0.36; 4.55	106	26	No
#10 post1–post2	0.8; 42.3	1.04; 41.56	51	27	Yes
#11 post1–post2	0.8; 59.1	0.98; 62.16	223	20	Yes
#12 post1–post2	0.8; 16.5	0.36; 16.21	52	27	Yes
#13 post1–post2	0.8; 2.5	0.36; 2.17	15	14	No
#13 post3–post4	0.8; 0.8	0.36; 0.36	152	25	No
#14 post1–post2	0.8; 3.4	0.76; 3.2	16	13	No
#15 post1–post2	0.8; 9.9	0.44; 9.48	85	27	Yes
#16 post1–post2	0.8; 1.2	0.65; 1.17	101	28	No
#16 post3–post4	0.8; 0.8	0.36; 0.54	157	28	No
#17 post1–post2	0.8; 0.8	0.41; 0.67	29	34	No
#17 post2–post3	0.8; 80.2	0.67; 80.41	63	28	Yes
#18 post1–post2	0.8; 4.7	0.36; 2.59	160	28	No
#19 post1–post2	0.8; 17.1	0.41; 18.62	51	28	Yes
#20 post1–post2	0.8; 1.4	0.36; 0.99	48	28	No
#21 post1–post2	0.8; 0.8	0.36; 0.78	101	40	No
#22 post1–post2	0.8; 0.8	0.36; 0.57	1656	30	No
#23 post1–post2	0.8; 0.8	0.36; 0.54	91	34	No
#24 post1–post2	0.8; 0.8	0.36; 0.36	238	27	No
#25 post1–post2	0.8; 0.8	0.36; 0.36	621	28	No
#26 post1–post2	0.8; 0.8	1.13; 2.7	305	31	No
#27 post1–post2	0.8; 0.8	0.95; 0.83	70	35	Yes
#28 post1–post2	0.8; 0.8	0.47; 0.44	57	48	No
#29 post1–post2	0.8; 0.8	0.36; 0.36	1205	20	No

All relapse events, except for ^#^ (diagnosed 42 days later) and ^##^ (diagnosed 4 days later), were diagnosed at Ct2. Ct1 and Ct2 represent the absolute values of chimerism at two consecutive time points, t1 and t2, where t2 immediately followst1. Abbreviations: STR, short tandem repeat; HCT, hematopoietic cell transplantation.

**Table 3:** Chimerism value (%) depending on the genotype utilized in the analysis of patient 20 post-1 HCT.

		Donor Genotype			
		Blind	Donor1	Donor2	Donor1 and Donor2
Recipient Genotype	Blind	0.09	99.89	0.11	0.09
	Recipient	0.14	58.95	0.15	0.15

**Table 4:** Diagnostic value of ΔC of STR-PCR and AllosSeq HCT for relapse detection at cut-off value > 0.236.

	Sensitivity (%)	Specificity (%)	PPV (%)	NPV (%)
STR-PCR	91.7	83.3	44.0	95.2
AlloSeq HCT	91.7	79.2	42.3	79.2

Abbreviations: STR, short tandem repeat; PPV, positive predictive value; NPV, negative predictive value.

**Table 5:** Comparison of the characteristics of AlloSeq HCT and STR.

Characteristic	AlloSeq HCT	STR
Lower limit of quantification	0.36% *	0.8% †
Lower limit of detection	0.22% *	0.8% †
Genetic markers	Single nucleotide polymorphism (1 bp)	Short tandem repeat (2–6 bp)
	Biallelic	Multiallelic
Number of markers	202	10
Input material	(0.625 ng/µL × 16 µL) = 10 ng	(1 ng/µL × 1.7 µL) = 1.7 ng
Minimal genotype needed	All participants minus one	All participants
%CV at 5% of chimerism	<5%	<20%
Applicability in multiple donors	Yes	Yes
Cost per test	Considerable	Relatively lower

Abbreviations: STR, short tandem repeat; bp, base pair; hematopoietic cell transplantation (HCT); CV, coefficient of variation. * Manufacturer claims, not verified by the authors. † Verified by the authors.

## Data Availability

Data are unavailable in order to protect patient privacy.
